# Very High‐Power Short‐Duration Ablation for Premature Ventricular Complexes From Sites With Suboptimal Catheter Stability

**DOI:** 10.1111/jce.70264

**Published:** 2026-01-20

**Authors:** Heather Wheat, Kelly Arps, Amrish Deshmukh, Michael Ghannam, Frank Bogun, Jackson J. Liang

**Affiliations:** ^1^ Electrophysiology section, Division of Cardiology University of Michigan Ann Arbor Michigan USA

## Abstract

**Introduction:**

While very high‐power short‐duration (vHPSD) ablation has been shown to be safe and effective for ablation of atrial fibrillation, the utility of vHPSD ablation for targeting premature ventricular complexes (PVCs) remains unclear. We aimed to describe our experience of PVC ablation using vHPSD ablation targeting areas with suboptimal catheter contact.

**Methods and Results:**

We included 8 patients (mean age 66.5 ± 11.3 years, 77% female gender, mean LV ejection fraction 52.8 ± 8.2%, baseline PVC burden 23.3 ± 10.1% [range 9–41%]) with PVCs originating from intracavitary structures [LV papillary muscle(s) (*n* = 7), RV papillary muscle (*n* = 1)] which were successfully eliminated with vHPSD ablation using a temperature‐controlled ablation catheter (QDOT‐MICRO; Biosense Webster, Irvine, California, USA) with lesions delivered at 90 W for 4 seconds using QMODE+ mode. Mean QMODE+ lesions delivered in each patient was 28 ± 15.1 with a mean total QMODE+ RF time of 112 ± 60.4 seconds. There were no procedural complications. Durable PVC suppression was confirmed on post‐ablation monitoring in all patients (mean post‐ablation PVC burden < 1% [range 0–2.3%]).

**Conclusion:**

Ablation with vHPSD using a temperature‐controlled radiofrequency ablation catheter can be safe and effective for PVC ablation in regions with poor catheter stability such as RV and LV papillary muscles.

1

Very high‐power short‐duration (vHPSD) ablation has been increasingly used for ablation of atrial fibrillation. For atrial ablation, vHPSD ablation has an excellent safety profile and may improve procedure efficiency by decreasing ablation time and procedure duration. One such modality includes QMODE+, which delivers 90 W for 4 s, as opposed to a more traditional QMODE lesion that offers up to 50 W for longer durations. While some variations using high‐power temperature‐controlled catheters have been shown to be effective for ventricular ablation, human studies remain limited [1, 2]. One prior study reported shorter procedure time with no difference in safety with vHPSD ablation compared to standard ablation of premature ventricular complexes (PVCs) [1]. The utility of vHPSD ablation for PVCs remains unclear and theoretically may be especially helpful in certain scenarios. We aimed to describe our experience of PVC ablation using vHPSD ablation targeting areas with poor catheter contact.

We included 8 patients (mean age 66.5 ± 11.3 years, 77% female gender, mean LV ejection fraction 52.8% ± 8.2%, 25% of whom were on antiarrhythmic drugs) who underwent PVC ablation at the University of Michigan between 10/2023‐8/2025. In all patients, targeted PVCs were successfully eliminated with vHPSD ablation using a temperature‐controlled ablation catheter (QDOT‐MICRO; Biosense Webster, Irvine, California, USA) with lesions delivered at 90 W for 4 s using QMODE+ mode. All patients had PVCs which originated from regions of poor catheter stability (LV papillary muscle(s) (*n* = 7), RV papillary muscle (*n* = 1)). Four (50%) patients had previously undergone at least one prior radiofrequency ablation session prior to the index procedure (two with one prior ablation session, two with two prior ablation sessions). In three of the four patients with at least one prior ablation session, acute PVC suppression was achieved during the prior procedure, but subsequent follow‐up event monitors confirmed late recurrence prompting referral for repeat ablation, while in one patient ablation was acutely unsuccessful due to suspected origin deep in the posteromedial papillary muscle.

During the index procedure, vHPSD QMODE+ lesions were delivered during the same procedure after clinical PVCs persisted despite ablation with standard QMODE ablation lesions in five patients; while in the remaining three patients only QMODE+ lesions were delivered. All successful QMODE+ ablation sites in the five patients who also had QMODE lesions were at sites also previously targeted with QMODE lesions. Mean QMODE+ lesions delivered in each patient was 28 ± 15.1 with a mean total QMODE + RF time of 112 ± 60.4 s. In each patient, PVCs were eliminated acutely with QMODE+ lesions and after PVC elimination, additional empiric lesions were delivered at and adjacent to the successful PVC suppression site for consolidation per operator discretion. Mean total fluoroscopy time was 4.4 ± 2.9 min and total procedure time was 2.3 ± 1.1 h. There were no procedural complications. Antiarrhythmic medications were discontinued in all patients post‐ablation. PVC burden assessment demonstrated reduction in median burden of targeted PVCs from 23.3% ± 10.1% (range 9%–41%) at baseline found on Holter monitor or extended cardiac event monitor, to < 1% (range 0%–2.3%) post‐ablation (with either 14‐day extended cardiac event monitoring or implantable loop recorder interrogation) after a mean period of 26.5 ± 11.7 days.

Ablation with vHPSD using a temperature‐controlled radiofrequency ablation catheter can be safe and effective for PVC ablation in regions with poor catheter stability such as RV and LV papillary muscles (Figure 1).

**Figure 1:**
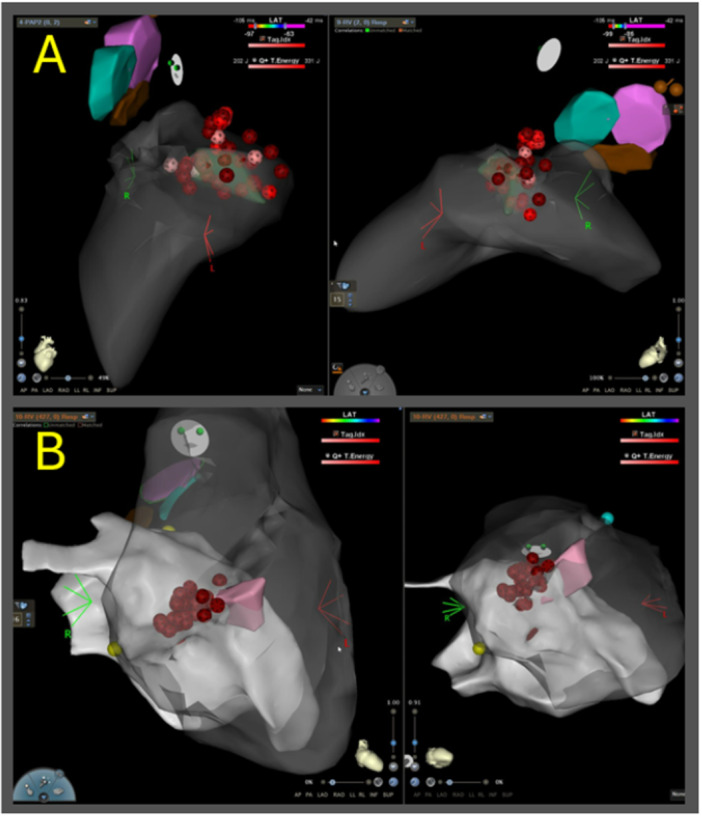
Example lesions delivered with vHPSD ablation targeting papillary muscles. Panel A shows vHPSD lesions delivered targeting an LV anterolateral papillary muscle PVC. Panel B shows vHPSD lesions delivered targeting an RV papillary muscle PVC.

Since one major goal during ventricular ablation is creation of deeper lesions and vHPSD typically creates shallower, wider lesions, utilizing vHPSD for ventricular ablation may seem paradoxical. However, in regions with poor catheter stability such as the papillary muscles, RV moderator band, false tendors, or along the tricuspid annulus, suboptimal catheter contact/stability may limit lesion formation due to inadequate conductive heating from the catheter tip. In these situations, the resistive heating component of the lesion may be more important and the ability to deliver more energy in the limited amount of time the catheter tip is in contact with the targeted tissue with vHPSD ablation may be beneficial. Additionally, in regions of poor stability, low‐power long lesions may be ineffective to create deeper lesions and it is possible that repeated administration with stacking of multiple short very high‐power lesions at these sites may result in deeper lesions, as has been demonstrated in swine models [3]. Importantly, due to shallow nature of lesions created with vHPSD ablation, arrhythmias originating from deep within tissue may not be able to be adequately targeted even with repeated vHPSD lesions at the same location. Strategies such as cryoablation or pulsed field ablation (PFA) have been effectively utilized to target PVCs from intracavitary structures [4, 5]. However, additional use of a cryoablation or PFA catheter after unsuccessful radiofrequency ablation may be associated with increased cost and procedure duration. In these situations, vHPSD ablation might be an effective and more cost‐effective alternative option to consider Figure 2.

**Figure 2:**
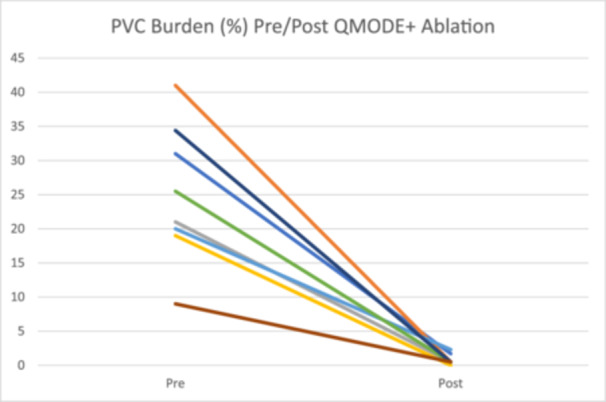
Change in PVC burden (%) in patients undergoing QMODE+ ablation.

## Conflicts of Interest

Dr. Liang has served as a consultant and received speaker honoraria from Abbott and Biosense Webster. The remaining authors have no relevant disclosures.
